# Use of Telemedicine to Improve Cognitive Functions and Psychological Well-Being in Patients with Breast Cancer: A Systematic Review of the Current Literature

**DOI:** 10.3390/cancers15041353

**Published:** 2023-02-20

**Authors:** Andreina Giustiniani, Laura Danesin, Rachele Pezzetta, Fabio Masina, Giulia Oliva, Giorgio Arcara, Francesca Burgio, Pierfranco Conte

**Affiliations:** IRCCS San Camillo Hospital, 30126 Venice, Italy

**Keywords:** oncology, telemedicine, telerehabilitation, psychological well-being, cognitive impairment, rehabilitation, cancer side effects

## Abstract

**Simple Summary:**

Breast cancer is one of the most frequently diagnosed cancers among women. This diagnosis is accompanied by many psychological implications as well as cognitive consequences due to both the cancer itself and cancer treatments. Recently, telemedicine approaches have been used to provide support to these patients. We conducted a systematic review to clarify the effectiveness of telerehabilitation for treating the cognitive and psychological difficulties of breast cancer patients. The literature suggests that telerehabilitation may represent a promising approach for breast cancer patients, but more studies are needed that address the role of telerehabilitation, especially for cognitive symptoms.

**Abstract:**

The diagnosis and side effects of breast cancer (BC) treatments greatly affect the everyday lives of women suffering from this disease, with relevant psychological and cognitive consequences. Several studies have reported the psychological effects of receiving a diagnosis of BC. Moreover, women undergoing anticancer therapies may exhibit cognitive impairment as a side effect of the treatments. The access to cognitive rehabilitation and psychological treatment for these patients is often limited by resources; women of childbearing age often encounter difficulties in completing rehabilitation programs requiring access to care institutions. Telemedicine, which provides health services using information and communication technologies, is a useful tool to overcome these limitations. In particular, telemedicine may represent an optimal way to guarantee cognitive rehabilitation, psychological support, and recovery to BC patients. Previous studies have reviewed the use of telemedicine to improve psychological well-being in BC patients, and a few have investigated the effect of telerehabilitation on cognitive deficits. This study systematically reviewed the evidence on the cognitive and psychological effects of telemedicine in BC patients. Current evidence suggests that telemedicine may represent a promising tool for the management of some psychological problems experienced by breast cancer patients, but more controlled studies are needed to clarify its effectiveness, especially for cognitive deficits. The results are also discussed in light of the intervening and modulating factors that may mediate both side effect occurrence and the success of the interventions.

## 1. Introduction

Breast cancer is the most frequently diagnosed cancer among women and, in spite of an increasing curability, still ranks as the primary cause of cancer-related death [1,2]. The number of women diagnosed with breast cancer is dramatically increasing, and survivors must face many challenges while they continue with their lives during and after cancer treatment. In particular, several studies have reported the occurrence of cognitive and psychological difficulties in breast cancer patients [3,4]. Indeed, a new generation of hormone therapies, targeted therapies, and immunotherapy have resulted in improved survival rates, but an increasing number of studies are reporting the impact of pharmacological therapies and of cancer itself on cognitive functions [5]. Cognitive difficulties have become a growing area of clinical concern, and they occur in about 25% of patients with cancer before pharmacological treatment, in about 75% of patients during treatment, and in about 35% of patients post-treatment [6]. There is considerable variability regarding the severity and the duration of cognitive impairment, with the most impaired cognitive domains usually being memory [7], processing speed [8], attention, and executive functions [9]. The variability may depend on factors such as the type of pharmacological therapy, the woman’s age and body mass index, and other disease-related biological factors such as inflammatory cytokine dysregulation, oxidative stress, DNA damage, or genetic polymorphisms and microvascular injury [10,11,12].

Psychological problems are common in breast cancer, and they have been shown to affect cognitive functioning [13]. Psychological difficulties are related to the fact that a breast cancer diagnosis is a stressful experience, causing significant psychosocial concerns such as marital problems or occupational difficulties [14], which vary along the disease trajectory. As is easily conceivable, patients may first develop depression and anxiety due to uncertainty as well as anger, sadness, and fear of death [15,16,17,18]. When the diagnosis comes during a phase of life in which women are developing their careers and having children, the psychological scenario is even worse [19]. In this context, in addition to anxiety and depression, women experience the emergence of negative feelings such as stress in significant relationships, sexual problems, separation anxiety, and fear of losing love, interest, support, and approval [20]. Furthermore, difficulties in managing health care while carrying on daily activities have important consequences for the quality of life, sleep quality, cognitive functioning, and even disease progression. Furthermore, the combination of cognitive and psychological problems may in turn influence women’s functioning at work, thus resulting in a vicious cycle that enhances negative emotions and cognitive impairment. Of note, while psychological dysfunctions are prominent in patients during breast cancer therapy and tend to decrease in survivors, cognitive impairment remains after treatment cessation [21]. In this situation, psychological support is pivotal immediately after the diagnosis and during treatment, and, in parallel, the rehabilitation of cognitive impairment becomes particularly relevant in both patients and survivors.

Long-term care and health management in breast cancer are easier when the patient is an active manager of their own health. A promising resource for promoting this attitude is telemedicine, a method of providing health care services using information communication technologies (ICTs). These technologies offer the opportunity to overcome the patient’s mobility problems and reduce costs for the national health system. To date, telemedicine has been widely used with promising results in terms of cost-effectiveness, in mental health, and in cognitive impairment [22,23,24,25]. Among the telemedicine approaches, telerehabilitation provides the delivery of rehabilitation programs through ICT. Telerehabilitation programs are used for home rehabilitation to improve motor, cognitive, or psychological dysfunctions with several advantages. Indeed, these programs also provide the opportunity to access rehabilitation for patients who cannot reach care centers, allow for the continuity of care over time and space, reduce care costs, and improve comfort for the patients, thus reducing the drop-out rates.

Telemedicine and telerehabilitation offer several advantages when used with breast cancer patients in which they have positive effects on both cancer-related and treatment-related psychological conditions [20]. Previous studies have reported improvements after telemedicine interventions in the quality of life (QoL), anxiety and depression, psychological distress, social functioning, and fatigue in patients with many different diseases including breast cancer, respiratory diseases, and diabetes [20,26]. However, very few studies have investigated the effects of telerehabilitation on cognition [21,22,23]. Overall, in spite of some inconsistencies [27], improvements in verbal fluency, processing speed, cognitive flexibility, memory, and working memory as well as in subjective cognitive functioning have been reported [28,29].

A previous review focused on the effect of telemedicine on mental problems experienced by breast cancer patients. The present study aims at expanding the current literature by reviewing studies on the effects of telemedicine on the psychological and cognitive difficulties experienced by breast cancer patients, and also by considering the role that specific mediating factors may play in the success rate of these techniques, highlighting future directions and needs. Because of the close relationship between the cognitive and psychological domains in breast cancer patients, it is important to clarify whether telemedicine and telerehabilitation can represent an effective approach to a comprehensive management of both psychological wellness and cognitive difficulties. In the present study, we therefore reviewed the research focused on the effect of telemedicine on psychological and/or cognitive functioning, providing an overview of factors that can play a role in the success rate of these interventions.

## 2. Materials and Methods

This study was conducted by following the Preferred Reporting Items for Systematic Reviews and Meta-Analyses (PRISMA) [30]. We systematically searched the following databases: Scopus, PubMed, and Embase from January 2000 to September 2022. We used the following keywords: “telemedicine”, “e-health”, “telerehabilitation”, “breast cancer”, “cognit*”, “psycholog*”. The six terms were combined using appropriate Boolean operators for search.

- To be eligible, studies had to meet the following criteria:
- Being concluded or planned randomized controlled trials (RCTs);
- Assessing the impact of telemedicine in patients treated for early breast cancer or breast cancer survivors after the completion of treatment;
- Reporting a cognitive test or psychological scales as primary or secondary outcomes;
- Using telemedicine for evaluation or rehabilitation;
- Being written in English;
- Being published in an English language journal after 2000.

Candidate studies were excluded when they were published in non-scientific journals, were not conducted on humans, used rehabilitation protocols other than telehealth, telemedicine, web-based therapy, online therapy, and did not use cognitive or psychological tests as the primary or secondary outcome. Duplicate studies were excluded using the Mendeley reference tool. Other reviews were inspected to extract possible eligible papers.

Six authors (AG, LD, FB, RP, FM, GO) independently screened the titles and abstracts of articles collected from the database search. Only articles meeting the inclusion criteria were selected. Any disagreement in study selection was discussed and resolved among all the authors.

The remaining articles were read by five authors (AG, LD, RP, FM, GO) who extracted relevant information following a modified version of the PICO guidelines: participants, methodology, comparisons, outcomes. Additional data on the sample’s demographic were extracted.

## 3. Results

**Figure 1:**
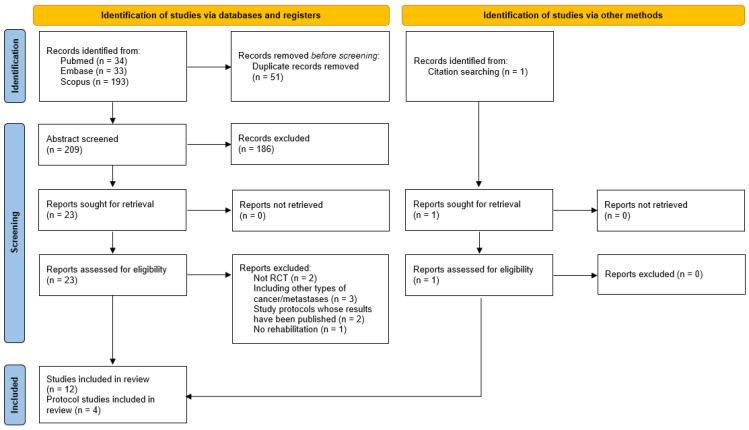
Search strategy used for the selection of studies included in the review.

Our search initially identified 260 records. After the removal of duplicates, 209 articles remained. During the abstract screening process, 186 studies were excluded and 23 studies were selected for the full text reading. During the full text reading, 16 records were determined to meet the inclusion criteria. Among these, four records were RCT study protocols (Figure 1).

### 3.1. Included Studies and Protocols-Sample Characteristics

From the reviewed studies, a total of 1754 participants constituted the sample. The women’s ages varied from 30 to 70 years, with only one study recruiting participants older than 70 years [31]. A total of 2898 participants were included in the study protocols (Table 1).

#### 3.1.1. Patients’ Cancer Stages in the Included Studies

Among the included records, one completed study recruited women with stage I–III primary breast cancer starting adjuvant therapy [31], and 11 studies recruited women during or after the completion of adjuvant endocrine therapy [27,29,32,33,34,35,36,37,38,39,40]. Moreover, four study protocols that planned to enroll patients who would start [41,42,43] or would complete [44] adjuvant or neoadjuvant therapies were included. Only one study and one study protocol reported some information on the molecular subtypes of breast cancer: the study from Zachariae [37] included 80% of patients with luminal tumors, and the protocol from Carlson [41] will include all subtypes except for HER2+ disease.

#### 3.1.2. Characteristics of the Telemedicine Programs and of the Control Conditions Applied in the Included Studies

Overall, the type of telerehabilitation employed in the included studies ranged from programs on physical fitness to psycho-education, cognitive behavioral therapy (CBT), mindfulness, and cognitive training. Similarly, the duration of these programs was heterogeneous, with most of the studies applying a 6–12 weeks of training.

Two studies used the E-CUIDATE program to improve QoL, pain, muscle strength, and fatigue [35,36]. This system consists of an interface in which patients perform tailored exercises consisting of a warm-up routine, resistance and aerobic exercise training, and a cool-down phase. The training consisted of three sessions per week. Each session lasted about 90 min and the entire training lasted 8 weeks.

Four studies applied CBT to improve sleep disturbances [37,40] and psychological well-being [38,39]. Oswald performed CBT through weekly 90-min educational group sessions for 6 weeks over a videoconference. The group received information about sleep education, hygiene, and medications as well as on cognitive restructuring [40]. Zachariae used the CBT program for an individual delivery of six psycho-educational themes: introduction and treatment rationale, sleep restriction, stimulus control, cognitive reconstruction, sleep hygiene, and relapse prevention [37]. Each theme was studied by participants in a 45–60 min session. Van den Berg used a system named BREATH to improve psychological well-being. The therapy included information, assignments (48 tasks), assessment of the difficulties, and educational video. BREATH is a pure self-help program without therapist contact [39]. The training lasted 16 weeks. Bandani-Susan performed a group online intervention with the aim of helping patients to manage cancer-related fatigue and body image and to encourage positive feelings [38]. The program consisted of 7 weeks of educational approaches, supervised physical activity, religious messages, cognitive therapy to improve body image and cognitive restructuring, and meditation.

Three studies provided psychoeducational material via telemedicine [29,31,34]. Specifically, in the Krzyzanowska study, patients were given a booklet for the management of common side effects of chemotherapy (e.g., nausea, vomiting, pain, fatigue) and received two structured follow-up calls during the cycle of chemotherapy to assess the frequency and severity of such symptoms [31]. In the study by Freeman and colleagues, the patients received four group sessions comprising didactic education on mind–body connection, mental imagery, and physiological processes followed by a discussion of the presented material [29]. Admiraal and colleagues asked participants to perform the ENCOURAGE program for 12 weeks, receiving psychoeducational material, coping strategies, and hyperlinks to address emotional and physical problems related to cancer [34].

One study applied a cognitive training program (i.e., HappyNeuron Pro) to improve cognitive functioning [27]. The program consisted of several tasks centered on attention, processing speed, learning, memory, working memory, and problem solving. Tasks were structured as a computerized game with different levels of difficulty. Each participant trained 30 min/day, 5 days/week, for 6 weeks. The program was performed online and accompanied by telephone and email-based support.

Lozano-Lozano combined in-person occupational sessions with the BENECA app, which provided nutritional recommendations to improve mood, cognitive functions, and physical functions in breast cancer survivors [32]. The BENECA app is a validated mobile health application that monitors the energy balance of individuals in terms of physical activity and diet and provides recommendations for improvement. The original BENECA program was extended by including exercises based on occupational therapy and on cognitive training. In another study by the same author, the same program was used to improve the quality of life.

With respect to the inclusion of a control group, four studies compared the telerehabilitation program with a waitlist group [27,37,38,40]. Three studies used patients undergoing standard care as a control group [31,34,39]. Three studies provided written recommendations to the control group on health and nutrition [39] and stress management [35,36]. One study also compared the efficacy of telerehabilitation with the delivery of the same program in person [29]. Finally, two studies used telerehabilitation as the control for an integrated approach combining telemedicine and face-to-face rehabilitation [32,33].

Concerning the study protocols, two studies will provide psychological and medical education on cancer [43,44]. One study protocol will use a mindfulness-based program to address psychological recovery in cancer patients [41]. Finally, Gonzales-Santos [42] will use a cognitive training program (E-OTCAT) focusing on attention, memory, and processing speed.

### 3.2. Effects on Cognition

Seven of the included studies investigated the effect of telerehabilitation programs on cognition after cancer treatment [27,29,33,35,36,41,42]. Five studies were original articles [27,29,33,35,36], and two were study protocols describing ongoing RCT [41,42].

Among the original articles, two studies exclusively considered objective neuropsychological assessment [33,35], two focused on subjective cognitive functioning [29,36], and the remaining one investigated both self-reported and objectively assessed cognitive functioning [27]. The two protocol papers will investigate self-reported and objectively assessed cognitive functioning [41,42]. All of the included studies focused on the cognitive domains most frequently reported to be impaired after cancer treatments such as executive functioning, working memory, attention, and information processing. One research study also examined verbal memory and learning as secondary outcomes [27].

Freeman and colleagues investigated the impact of an imagery-based group intervention delivered through telemedicine on self-reported cognitive functioning, which was assessed with the cognitive subscale of the Functional Assessment of Cancer Therapy (FACT-Cog version 2) at baseline, 1-, and 3-months follow-up. Results revealed an improvement in subjective cognitive functioning after both telerehabilitation and live-delivered interventions compared to the waiting list control group. The improvement detected in cognition was considered clinically significant and was maintained at the 3-month follow-up [29].

Similarly, Galiano-Castillo and colleagues investigated the effect of an 8-week Internet-based, tailored physical exercise program (E-CUIDATE) on the quality of life, pain, muscle strength, and fatigue in patients who had completed adjuvant therapy compared to a control group receiving basic recommendations on physical exercise [28]. The European Organization for Research and Treatment of Cancer Quality-of-Life Questionnaire Core 30 was administered, which assesses various aspects of the quality of life and includes two items about self-reported cognitive functioning. The authors found an improvement in self-reported cognitive functioning for the telerehabilitation group compared to the control group, which was maintained at a 6-month follow-up.

In a secondary analysis of data from this previous study, Galiano-Castillo found mixed results. The authors examined the efficacy of the E-CUIDATE in improving the functional and cognitive abilities in breast cancer survivors. In this case, cognitive functioning was assessed by the objective measures of short-term memory, attention, information processing, and mental flexibility. A lasting improvement was found for the group receiving telerehabilitation only in information processing and not in other cognitive domains [35].

In a recent study, Lozano-Lozano and colleagues examined the efficacy of the BENECA app combined with in-person occupational sessions on cognition, mood, and physical function. The authors found that selective attention (assessed with the Trail Making Test [45]) was significantly higher after the combined intervention compared to the control group that received the BENECA app alone, with a moderate-to-large effect size for TMT-A, working memory, and processing speed (assessed through the WAIS-IV), at 2 and 6 months after the intervention [33].

Damholdt found the opposite result. Indeed, the author reported no changes in working memory and attention in a group of breast cancer patients receiving a web-based telerehabilitation program with telephone support compared to a waiting list control group. The primary outcome of cognitive functioning was assessed with the Paced Auditory Sequence Test (PASAT [46]), a working memory and attentional span test. Of note, in this study, the authors compared the differences between self-reported and objectively measured cognitive functions. Other neuropsychological measures were verbal learning, working memory, and executive functioning indices. No statistical changes were found in the former nor in the latter. However, a small improvement was found in verbal learning and in the working memory tests post-intervention and at the 5-month follow-up [27].

#### Effects on Cognition: Study Protocols

Two of the included studies were RCT study protocols [41,42] aiming at investigating the efficacy of two different telemedicine approaches in preventing and mitigating the cognitive and psychological (i.e., anxiety and depression) and other common consequences of breast cancer and chemotherapy such as fatigue, pain, sleep disturbances, nausea/vomiting, and quality of life. Regarding cognition, both studies will investigate self-reported and objective cognitive functioning (see Table 1).

The more recent study designed an RCT protocol aiming to investigate the efficacy of videoconference-based cognitive adaptive training (eOCTAT) in preventing cancer-related cognitive impairment in patients with breast cancer undergoing chemotherapy [42]. Participants will be randomized to either the experimental group that will receive the e-OTCAT program for 12 consecutive weeks from the beginning of chemotherapy or the control group, which will receive an educational handbook and the usual care. Assessment will focus on cognitive functioning and psychological distress, fatigue, sleep disturbance, quality of life, and occupational performance will be investigated. Subjective cognitive complaints will be measured with the Cog-FACT [47]. Assessments will be conducted before chemotherapy (baseline) and at 6 and 12 months after the baseline.

Similarly, Carlson and colleagues designed an RCT aiming to determine the efficacy of an online mindfulness group for breast cancer patients during chemotherapy in 12 real-time interactive weekly sessions [41]. In this case, the online intervention will be aimed at primarily managing fatigue and other common post-chemotherapy symptoms (i.e., sleep disturbance, pain, nausea/vomiting, mood, stress, quality of life), whereas cognition will be explored as a secondary outcome. Patients will be randomized to the experimental group or a waiting list control group, with assessments at four time points: baseline (pre-chemotherapy), post-rehabilitation, post-chemotherapy, and 12 months post-baseline. Self-reported cognitive functioning will be assessed with the Cog-FACT [47], while objective cognitive functioning will be assessed through the Sustained Attention to Response Task, a computer-based go/no-go task designed to measure working memory, sustained attention, and impulse/inhibitory control. If effective, both of these ongoing RCTs will provide support and more evidence about the implementation of telemedicine approaches in oncological care.

### 3.3. Psychological Effects

#### 3.3.1. Quality of Life

Three of the included studies investigated the clinical implications and benefits of telemedicine for the general QoL of breast cancer patients (see Table 1).

Among these, Admiraal and colleagues reported no differences between psycho-educational approaches and standard care in problem-solving strategies and other psychological outcomes measured at the baseline, 6, and 12 weeks (see Table 1) [34]. An unplanned subgroup analysis showed that in clinically distressed patients (n = 57), participation in the web-based program resulted in more optimism and control over the future at 12 weeks than the control group patients, suggesting that the lack of effects between groups might be due to some patients being unable to further increase their optimism.

Lozano-Lozano and colleagues [32] compared the effect of the mobile BENECA app combined with a supervised rehabilitation program versus the BENECA app alone. In this study, patients were assessed with questionnaires at the baseline, 2-months post-intervention, and 6-month follow-up. Both rehabilitation programs improved the QoL, with global QoL significantly better with the BENECA app plus rehabilitation than with the BENECA program alone, with a moderate-to-large effect size. The clinically significant effect on QoL was maintained during the follow-up.

Galiano-Castillo and colleagues compared a telerehabilitation group (8-week Internet-based intervention) with a control group at the baseline, after 8 weeks and at a 6-month follow-up. Results showed that the telerehabilitation group improved regarding the QoL, which was maintained during the follow-up check [36].

As shown by Freeman and colleagues, an aspect that seems crucial in the effectiveness of treatment is the web-mediated interaction with a therapist who actively interacts with the patient [29]. The aim of this study was to compare the effects of an imagery-based behavioral intervention delivered live or via telemedicine compared to a waitlist control on the QoL of breast cancer survivors. Their system consisted of videoconferencing software that enabled the therapist to view and interact with the patient. Their results revealed the beneficial effects of the intervention for improving QoL in cancer survivors. Remarkably, it seems that involvement in the telemedicine-delivered intervention did not result in different outcomes compared to the intervention delivered with a therapist physically present.

#### 3.3.2. Sleep

Several authors developed web-based interventions for sleep disturbances on the basis of CBT with the aim of enabling patients to cope with problems related to the diagnosis or the administration of cancer treatments. Following the application of web-based CBT programs to treat sleep disturbance, Zachariae and colleagues [37], and later Oswald and colleagues, proposed two RCTs to assess the efficiency of this type of rehabilitation in breast cancer survivors [40] (see Table 1).

In the former, women with breast cancer who experienced clinically significant sleep disturbance were randomly allocated to a CBT program or to a waitlist control group. Insomnia severity, sleep quality, and fatigue measures were collected at the baseline, post-intervention (9 weeks), and follow-up (15 weeks). Breast cancer survivors following the CBT program showed reduced insomnia severity and improved the overall sleep quality. Indeed, significant effects were found for all sleep-related outcomes from pre- to post-intervention. Furthermore, improvements were maintained for outcomes measured at follow-up. Similarly, in the study of Oswald and colleagues, the breast cancer survivors were randomized to a CBT group to treat insomnia or to a waitlist control for 6 weeks. Results showed that post-intervention, there were medium-to-large group differences for secondary outcomes of interest such as insomnia symptoms, sleep disturbance, and sleep efficiency, with CBT showing a preliminary efficiency compared to the control group. In addition, group differences after intervention indicated that participants who reported clinically significant symptomatology all favored the eHealth CBT condition, with small/medium to medium/large effect sizes. Limitations of this study included the use of a waitlist control group instead of a robust attention-control comparison.

#### 3.3.3. Fatigue

Besides investigating insomnia and general sleep quality, Zachariae and colleagues also assessed the levels of fatigue of groups of women with breast cancer experiencing clinically significant sleep disturbance, finding benefits in terms of reduced fatigue in those who followed CBT, compared to the waitlist [37].

In a recent study conducted by Bandani-Susan and colleagues, the efficacy of a mobile health educational intervention in improving cancer fatigue and body image was investigated [38]. Results showed that the mobile intervention improved the levels of fatigue and body image among breast cancer survivors. Limitations concerning the small sample size were highlighted (see Table 1).

Furthermore, Galiano-Castillo and colleagues demonstrated that the telerehabilitation intervention improved aspects of the QoL compared to the control group, and that it improved the general levels of fatigue perception. Of note, these improvements were maintained at the follow-up [36].

#### 3.3.4. Anxiety, Depression, and Distress

A few studies have investigated the effects of using mobile interventions on improving mood, depression or anxiety feelings, and distress in patients with breast cancer.

Similar to previous interventions, van den Berg and colleagues developed a web-based self-management intervention based on the principles of CBT to reduce distress and improve empowerment [39]. Patients could choose to access a wide range of materials (assignments, self-assessments, and videos) that were released on a website. Since the intervention was a self-management program, it did not require real interaction or contact with a therapist. The findings indicated that the intervention contributed to reducing the level of distress in patients without affecting empowerment.

Finally, studies investigating the effects on anxiety and depression did not report any significant improvement after treatment delivered through telemedicine [31].

#### 3.3.5. Pain

We only found one study investigating the effect of telerehabilitation on pain perception. In this study, it was shown that the telerehabilitation group improved regarding aspects of pain severity and pain interference; the results for the pain interference effects, but not pain severity, were maintained during the follow-up [36].

#### 3.3.6. Psychological Effects: Study Protocols

Four of the included studies were RCT study protocols, therefore the results are not available yet.

In two of these studies, the interventions were developed to promote, through interactive programs of telerehabilitation, a healthy lifestyle, together with other typical outcomes such as QoL, fatigue, anxiety, and depression [42,44].

Carlson and colleagues plan to apply a mindfulness-based intervention. This program will be administered during chemotherapy in 12 real-time interactive weekly sessions with the principal aim of managing fatigue, and in addition to this primary outcome, insomnia, pain, nausea/vomiting, mood, distress, and QoL. Crucial in this intervention are the recommendations for patients to practice mindfulness exercises for 30–45 min per session [41].

Lidington and colleagues will explore the effectiveness of a mobile application for self-monitoring symptoms and managing care in patients with breast cancer (see Table 1) The authors will investigate whether using the application may affect QoL, health status, and distress [43].

González-Santos and colleagues are conducting an RCT aimed at investigating the effects of a videoconference cognitive-adaptive training (e-OTCAT) for 12 weeks from the beginning of chemotherapy. Outcomes will be the cognitive function, psychological distress, fatigue, sleep disturbance, QoL, and occupational performance, measured at the baseline, after 12 weeks, and 6 months of post-randomization. The authors are interested in understanding whether the telemedicine approach can prevent cognitive impairments and other effects of cancer and its treatment [42].

## 4. Discussion

The aim of the present paper was to systematically review the literature on the current telemedicine interventions applied to improve psychological and/or cognitive functions in breast cancer patients both during and after pharmacological therapies. Only RCT studies were considered to define the state-of-the-art, and the study protocols were included to shed light on possible future paths and fields of investigation in both clinical and scientific practices. In particular, we were interested in understanding whether telemedicine can represent a valuable option for the rehabilitation of breast cancer patients as it allows for the combination of both psychological support and cognitive rehabilitation, which are two crucial needs of breast cancer patients and survivors.

Contrary to our expectations, only a few studies have investigated both the cognitive and psychological effects of telerehabilitation. Among these, only one used a telerehabilitation program with the specific aim of improving both psychological and cognitive functions [32]. The other studies were aimed at improving either the cognitive or the psychological effects. Overall, these three studies highlight that combining telerehabilitation with the presence of a therapist led to the best results, whereas not explicitly providing participants with the opportunity to contact the therapist in case of need led to the worst results, namely, inconsistent improvement in working memory tests with no changes in all the other trained domains [27]. However, in this latter study, an important issue that may have limited the significance of the results was that the neuropsychological assessment was conducted via telephone.

Of note, in all of the included studies but one [27], cognitive performance was measured as a primary or secondary outcome after treatments that were only partially focused on cognitive tasks and mostly based on occupational therapy, psycho-educational approaches, physical activity, and body exercises.

Overall, some considerations arose from these studies. First, in each study, cognitive domains were assessed with many different neuropsychological tests, which may have distinct levels of sensitivity to cognitive impairments, thus leading to heterogeneous and variable findings. Similarly, the methodology used for the neuropsychological assessment varied among studies, with some studies reporting a face-to-face assessment and other studies reporting telephonically conducted neuropsychological interviews. To complicate the matter further, information was generally lacking about whether rehabilitation programs were individualized based on the patients’ specific deficits. This aspect is crucial for cognitive rehabilitation to have meaningful clinical results and should be addressed when considering experimental findings. Another concern was that cognitive deficits were reported as either objectively measured or self-assessed by the patients. There can be a great discrepancy between deficits measured by a professional and deficits reported by the patient, with the latter being even more susceptible to intervening psychological factors. More studies should compare the effects of telerehabilitation in terms of the perceived and objectively measured cognitive impairment to clarify this issue. Furthermore, there was wide variability with respect to the employed telerehabilitation programs, with the one used by Lozano and colleagues being the most effective, which induced a stable improvement in all the studied domains [32]. On the other hand, in this study, we could not exclude that the strong presence of face-to-face support for the patients during the training might have played a role in modulating the observed results.

Finally, an important consideration is that to date, only a few studies using telemedicine have focused the intervention on both cognitive and psychological factors, thus suggesting that the interaction between these aspects has not been fully addressed. Indeed, in many of the included studies, the observed results on the psychological and cognitive factors were maintained separately. In contrast, even with a lack of effect, the role of one or the other should be considered and discussed as these two aspects often influence each other [48,49].

In the present study, we found that the domain that benefitted the most from telemedicine is probably the QoL. Indeed, an improvement in QoL was reported in almost all of the reviewed studies. Of note, the improvement substantially remained at follow-up, that is, it remained, despite cancer progression and treatment side effects. For instance, a long-lasting (i.e., 6 months) improvement in QoL was observed with long (i.e., 8 weeks) treatment durations [32,35]. Unfortunately, other psychological aspects were not investigated in these two studies.

Overall, the research findings prove that telemedicine practices have an impact on the QoL of breast cancer patients. Among the psycho-education approaches, BENECA [32] and imagery-based behavioral interventions [29] were the most effective. The lack of effect reported by only one of the studies reviewed here [34] might have depended on the general ineffectiveness of problem-solving oriented programs, even when targeted at the patients’ needs. However, other factors may have played a role such as the cancer stages and the different symptoms experienced by patients due to different pharmacological treatments.

Sleep problems are often a major complaint of breast cancer patients, and they are usually treated on the basis of CBT. We found only two studies that applied web-based CBT, reporting that it may be an efficacious treatment option for breast cancer survivors with robust and clinically relevant effects. Similarly, telemedicine has been proven to be effective in reducing fatigue when related to sleep difficulties [37,40]. However, more studies are needed to replicate these promising and encouraging results.

Conversely, less encouraging were the results with respect to anxiety and depression. Indeed, where a general reduction in distress levels was reported by previous studies [39], no effects were reported on the anxiety and depression levels [31]. These findings are in contrast to a previous review reporting that technology-based interventions were effective for depressive symptoms and anxiety experienced by women with breast cancer [26]. The discrepancy between this previous study and our findings is probably due to the fact that in the former, RCTs were included as well as studies focusing on specific ethnic populations. On the other hand, our results were limited by the low number of included studies. Therefore, more controlled studies are needed to clarify this issue.

Pain is another common side effect of both surgery and hormonal therapies in breast cancer patients. In particular, after breast cancer surgery, the pain levels experienced by patients are high, so they often use opioids for pain reduction. Similarly, patients receiving aromatase inhibitors generally report arthralgia and myalgia [36]. The studies included in the present review suggested that telemedicine-based interventions, by teaching patients strategies to manage pain, could be useful to reduce pain perception and opioid use. Therefore, these interventions should be integrated in standard programs to enhance recovery and complement medical treatments. However, these results were limited, being based on only two studies. More studies are needed to investigate the effect of telemedicine on pain, taking into account other factors that are reported to influence pain perception such as menopause [36].

However, another consideration concerns the lack of a gold standard with respect to the use of telemedicine in breast cancer patients. Indeed, clarification is needed as to which program would be most effective based on the patients’ specific needs.

These programs should be targeted specifically at psychological and/or cognitive functioning and should follow a precise cognitive and psychological assessment. Overall, based on the current literature, reliable cognitive telerehabilitation should include cognitive tasks as well as psycho-educational intervention to train cognitive functioning and provide patients with information related to the treatment side effects. This training should not last less than 3 weeks, and ideally, it should be performed until the end of the breast cancer therapy and include a follow-up evaluation. Similarly, the current literature suggests that to maximize the benefits of psychological interventions, programs should be based on CBT and include both individual and group sessions in which patients ideally are provided with information about their status as well as cognitive restructuring. In this case, the remote on-demand presence of a therapist will be pivotal. Regarding cognitive training, psychological support should be provided from the diagnosis to the end of chemotherapy.

There are many other difficulties that patients experience after a breast cancer diagnosis such as sexual problems [50], which can benefit from a telemedicine approach. Studies are needed investigating this field.

## 5. Considerations on Mediating Factors and Unmet Needs

Understanding the factors that may contribute to the development of cognitive and psychological problems in patients treated for breast cancer was behind the purpose of the present review. However, factors involved in the emergence of such deficits may also contribute to their maintenance and can affect the success rate of both psychological and cognitive telemedicine-based interventions. These factors may be related to (1) cancer subtypes (luminal, HER2+, triple negative) and treatments (chemotherapy, hormonal therapies, biological therapies); (2) patient lifestyles; (3) biological factors (i.e., inflammation, oxidative stress, DNA damage and repair, genetic susceptibility, decreased telomere length and cell senescence [51]); (4) psychological factors and distress levels; (5) genetic variations; and (6) demographic factors. First, stronger cognitive dysfunctions have been reported for breast cancer patients exposed to both chemotherapy and hormone therapy than for patients exposed to chemotherapy only [52]. This observation holds true, especially for post-menopausal women [53]. The pivotal role played by estrogens in cognitive performance and psychological aspects might explain the potential negative effect of hormone therapies on both the cognition and psychological well-being of breast cancer patients. Similarly, the estrogen depletion induced by hormonal therapies might account for the possible reduced effects of concurrent cognitive rehabilitation and psychological treatments.

Several biological factors may play a role in both the occurrence of cognitive symptoms and in the effect of cognitive rehabilitation. Systemic inflammation can cross the blood–brain barrier and have a deleterious effect on the central nervous system [54], thus inducing cognitive impairment [55]. Anticancer treatment-induced cytokine storms may hamper or annul the beneficial effects of treatment.

Furthermore, elevated levels of C-reactive protein reflecting chronic inflammation may also play a role in cognitive problems [56], and the levels of this protein, together with other biological factors, may impact the efficacy of telerehabilitation and psychological telemedicine.

Genetic factors have been suggested to play a role in cognitive dysfunctions. For instance, variants of genes encoding apolipoprotein E (ApoE) and catechol-O-methyltransferase (COMT) have both been associated with age-related cognitive decline in the general population [57].

Finally, demographic factors may contribute to cognitive impairment and psychological symptoms. In particular, age (with older patients who are likely more vulnerable to pre- and post-treatment cancer-related side effects), race, and education have been shown to be associated with the presence of impairment in breast cancer patients [58]. Similarly, these factors may affect the success of the rehabilitation.

Psychological and emotional stress can alter the sympathetic nervous system and, in turn, the immune system [59]. In other words, psychological distress consequent to cancer treatment and side effects may trigger biologic alterations in the brain. These modifications may create long-term homeostatic changes that are responsible for the neuroplastic alterations leading to cognitive dysfunctions. Neuroplasticity is a crucial process underlying the effects of cognitive rehabilitation [60]. Altered or absent neuroplastic processes prevent training-related cognitive changes and may be responsible for the lack of improvement observed after cognitive rehabilitation in some of the studies reviewed here. Similarly, after breast cancer diagnosis, patients may experience post-traumatic growth [61], an experience that should be monitored during psychological treatment because of the confounding impact it can have on the effects of psychological telemedicine interventions. Finally, we must acknowledge that nowadays, breast cancer diagnosis includes several pathological conditions with very different natural histories, treatments, and prognoses. Each of these factors may affect the patients’ psychological conditions, needs, and responses to interventions, and should therefore be addressed in future studies.

## 6. Conclusions and Future Perspectives

In general, the current literature highlights the need for more controlled studies that are designed based on the general guidelines on breast cancer [62]. These guidelines should be updated in order to consider both the cognitive and psychological difficulties exhibited by breast cancer patients. With respect to cognitive evaluation and rehabilitation, a standard neuropsychological assessment including ad hoc testing as well as a standard procedure for test administration is currently lacking. Additionally, a distinction should be made between the self-assessment and objectively measured deficits as these are both important but not directly comparable. Similarly, with respect to psychological concerns, novel telemedicine-based approaches are needed that focus on specific interventions related to the wide range of difficulties experienced by breast cancer patients, namely, depression and anxiety, and the patients’ demographics should be the focus of new RCT studies. Along these lines, further studies should target both cognitive and psychological factors with specific telemedicine-based protocols that also consider the molecular classification and new standard of therapy. Moreover, it must be highlighted that while most studies have shown that psycho-educational approaches improve cognitive functions, future studies should apply dedicated cognitive telerehabilitation programs.

In conclusion, evidence is promising with respect to the use of telemedicine in breast cancer patients; however, current evidence also poses the need for more controlled studies to clarify the effectiveness of telemedicine, especially for cognitive deficits, but also for psychological problems (e.g., anxiety and depression).

## Figures and Tables

**Table 1:** Characteristic of the (**A**) included studies and (**B**) included study protocols [21,23,25,26,27,28,29,31,32,33,34,35,36,37,38].

(A)									
Author and Year	Sample Size	Age	Cancer Stage	Treatment	Type of Telemedicine Applied	Control	Duration	Outcome Variables	Results
Admiraal et al., 2017 [27]	139	C: 53.2 ± 8.5; T: 53.1 ± 9.8	I–III	Completed curative-intent primary treatment (surgery + chemotherapy) within the past six months	ENCOURAGE program: Psycho-education, problem-solving strategies for coping	Standard care	12 weeks	EORTC, QoL_Questionnaire, optimism and control over the future scale, Dutch Distress Thermometer, 47-item problem list	Increased optimism and control over the future
Bandani-Susan et al., 2021 [31]	38	46.34 ± 9.96 (C: 45.89 ± 7.64; T: 46.79 ± 12.28)	NA	Ongoing chemotherapy, radiotherapy, hormone therapy and/or brachytherapy	Educational messages, cognitive behavioral therapy for improving body image, and cognitive restructuring	Waitlist	49 days	CFS; Body Image Concern Inventory	Significant difference in quality of life (body image and cancer-related fatigue)
Damholdt et al., 2016 [21]	157	C: 54.56 ± 8.74; T: 54.98 ± 8.51	0-III	Ongoing chemotherapy, radiotherapy, or hormonal therapy	HappyNeuron Pro: Web-based program for cognitive training focused on six cognitive domains (attention, processing speed, learning, memory, working memory, problem-solving)	Waitlist	6 weeks	PASAT, RAVLT, Digit Span Forwards, Digit Span Backwards, Digit Ordering, Letter Fluency Test, 20 Questions Test, Cognitive Estimation Task, BDI, Whitely-7, SCL-ANX4 from Symptoms Checklist-92, self-reported benefit from the training	Improvements in verbal learning and working memory
Freeman et al., 2014 [23]	102	C: 55.28 ± 7.90; LD: 55.44 ± 8.08; T: 55.57 ± 9.88	0-IV	Completed main treatment from at least 6 weeks	Videoconference groups, education on the mind–body connection and on mental imagery	Live delivered (LD) psycho-educational groups; waitlist	5 weeks	Medical Outcomes Study survey (SF-36); FACT-B; FACIT-Fatigue Scale; FACT-Cog; Functional Assessment of Chronic Illness Therapy; Brief Symptom Inventory-GSI; Pittsburgh Sleep Quality Index	Improvement in fatigue, cognitive dysfunction, sleep disturbance, and health-related and breast cancer-related QoL for LD and TD groups compared to waitlist
Galiano-Castillo et al., 2016 [29]	81	C: 49.2 ± 7.9; T: 47.4 ± 9.6	I-III	Completed adjuvant therapy except hormone treatment	e-CUIDATE: Online system for the remote administration of physical exercises focused on resistance, aerobic, mobility, and stretching	Written recommendation on stress management, physical fitness	8 weeks	EORTC QLQ-C30; Brief Pain Inventory short form; Piper Fatigue Scale-revised	Improvements in global health status, pain severity, interference, and total fatigue
Galiano-Castillo et al., 2017 [28]	81	48.30 ± 8.80	I-III	Completed adjuvant therapy except hormone treatment	e-CUIDATE: Online system for the remote administration of physical	Written recommendation on stress management, physical fitness	8 weeks	6 Min Walk Test; Auditory Consonant Trigrams; TMT	Improvements in functional capacity and cognitive functioning maintained at 6 months
Krzyzanowska et al., 2021 [25]	561	55.7 *	I-IV	Starting adjuvant or neoadjuvant chemotherapy	Symptom Self-Management Booklet-patient edition + follow-up calls to address the presence of chemotherapy side effects	Standard care	Duration of chemotherapy cycle	NCI PRO-CTCAE; Stanford self-management self-efficacy scale; European quality-of-life; Patient Health Questionnaire 9; VAS; Generalized anxiety disorder; FACT-B	No differences in self-efficacy, anxiety, or depression
Lozano-Lozano et al., 2020 [34]	80	C: 49.76 ± 8.42; T: 53.40 ± 8.66	I-III	Completed adjuvant therapy except hormonal therapy	BENECA mHEALTH + rehabilitation: Supervised used of the BENECA app occupational therapy focused on reduction of fatigue and improvement of processing speed, working memory, and attention	BENECA mHEALTH: App for recommendation on physical activity and nutrition	8 weeks	EORTC- QoL Questionnaire; Breast Cancer-Specific Quality of Life Questionnaire;	Improvement in QoL, maintained after 6 months
Lozano-Lozano et al., 2022 [26]	80	C: 49.76 ± 8.42; T: 53.40 ± 8.66	I-III	Completed chemotherapy, could continue therapy with hormone	BENECA mHEALTH + rehabilitation: Supervised used of the BENECA app for recommendation on physical activity and nutrition; occupational therapy focused on reduction of fatigue and improvement of processing speed, working memory and attention	BENECA mHEALTH: App for recommendation on physical activity and nutrition	8 weeks	TMT; WAIS; Hospital Anxiety and Depression Scale; Brief pain inventory; Piper Fatigue Scale-revised; 6 Min Walk Test	Greater improvements in selective attention, working memory, and processing speed, anxiety, and functional capacity at 8 weeks and 6 months. Fatigue perception and pain were also improved
Oswald et al., 2022 [33]	30	58.44 ± 9.22 (C: 56.90 ± 8.91; T: 59.98 ± 9.58)	NA	Completed primary cancer treatment	CBT-I: Group sessions of cognitive behavioral therapy focused on sleep education, sleep restriction, stimulus control, sleep hygiene, and sleep medications, cognitive restructuring, and relapse prevention	Waitlist	6 weeks	Treatment Perceptions Questionnaire; 7-item Insomnia Severity Index; Pittsburgh Sleep Quality Index	Improvements in insomnia symptoms, sleep disturbance, and sleep efficiency compared to the control group
van den Berg et al., 2015 [32]	150	C: 50.18 ± 9.15; T: 51.44 ± 8.30	NA	Completed primary cancer treatment (surgery plus adjuvant chemotherapy and/or radiotherapy)	BREATH: Cognitive behavioral therapy online self-help program for the four phases of adjustment to breast cancer (looking back, emotional processing, strengthening, and looking ahead)	Standard care	16 weeks	Symptom Checklist-90; Cancer Empowerment Questionnaire; Hospital Anxiety and Depression Scale; EORTC Quality of Life Questionnaire Core 30; Distress Thermometer; Illness Cognitions Questionnaire; Remoralization Scale; Mastery Scale; Positive Adjustment Questionnaire; Self-Efficacy Scale; Cancer Worry Scale; Cancer Acceptance Scale; Checklist Individual Strength-Fatigue; Openness to discuss hereditary cancer in the family; Big Five Inventory	Reduced distress. Not persistent at follow-up
	255	53.1 ± 8.8 (C: 52.9 ± 8.9; T: 53.2 ± 8.8)	I-III	Ongoing chemotherapy, radiotherapy, or endocrine therapy	SHUTi: Automated interactive cognitive behavioral therapy for insomnia focused on sleep restriction and stimulus control, cognitive restructuring, sleep hygiene, and relapse prevention	Waitlist	6 weeks	Sleep diary; Insomnia Severity Index; Pittsburgh Sleep Quality Index; Functional Assessment of Chronic Illness Therapy for Fatigue	iCBT-I groups showed improvements in sleep-related outcomes which were maintained at 15 weeks follow-up
**(B)**									
**Author and Year**	**Sample Size**	**Age**	**Cancer Stage**	**Treatment**	**Type of Telemedicine Applied**	**Control**	**Duration**	**Outcome Variables**	
Carlson et al., 2019 [35]	178	>18	I–III	Scheduled for chemotherapy	Mindfulness-based cancer recovery, online group	Standard care, waitlist	12 weeks	Brief Screen for Cognitive Impairment; Functional Assessment of Chronic Illness Therapy—Fatigue; Pittsburgh Sleep Quality Index; Brief Pain Inventory; Osoba Nausea and Vomiting Module; FACT—General; Calgary Symptoms of Stress Inventory; Profile of Mood States—Short Form; FACT—Cog; Sustained Attention to Response Task; blood counts	
González-Santos et al., 2022 [36]	98	>18	I–III	Scheduled for chemotherapy	e-OTCAT program: Cognitive training using paper-pencil exercises and the NeuroNation mobile app	Standard care, provision of educational handbook on cancer treatment side effects	12 weeks	FACT-Cog; TMT; WAIS; Hospital Anxiety and Depression Scale; Piper Fatigue Scale-Revised; Pittsburgh Sleep Quality Index; EORTC-Quality of Life Questionnaire Core; Canadian Occupational Performance Measure	
Krusche et al., 2019 [38]	2500	>18	NA	Finished primary cancer treatment within prior ten years	Renewed: Software addressing four main areas (physical activity, stress reduction, diet improvement, weight loss)	Standard care, provision of educational resources	NA	QoL; fear of relapses; anxiety and depression; website satisfaction and usage	
Lidington et al., 2020 [37]	122	>18	Early stage	Non-specified anticancer treatment	OWise: Online tool offering tailored medical information, medical terms glossary, useful links to local resources, tracking tool for symptoms, and a consultation recording device		Standard care, provision of educational resources	NA	Hospital Anxiety and Depression Scale; EORTC QLQ C-30; EuroQol 5-Dimension 5-Level questionnaire (EQ-5D-5L)

Notes for (A): Quality of Life (QoL); Cancer Fatigue Scale (CSF); Beck Depression Inventory (BDI); Global Severity Index (GSI); Rey Auditory Verbal Learning Test (RAVLT); National Cancer Institute Patient Reported Outcomes version of the Common Terminology Criteria for Adverse Events (NCI PRO-CTCAE); Trail making test (TMT); Visual Analogue Scale (VAS); Functional Assessment of Cancer Therapy for Patients with Breast Cancer (FACT-B); European Organization for Research and Treatment of Cancer (EORTC); Wechsler Adult Intelligence Scale (WAIS); * Median. Notes for (B): Functional Assessment of Cancer Therapy for Patients with Breast Cancer (FACT-cog).
